# *Brucella pinnipedialis* in hooded seal (*Cystophora cristata*) primary epithelial cells

**DOI:** 10.1186/s13028-016-0188-5

**Published:** 2016-01-25

**Authors:** Anett Kristin Larsen, Jacques Godfroid, Ingebjørg Helena Nymo

**Affiliations:** 1 Department of Arctic and Marine Biology, Faculty of Biosciences, Fisheries and Economics, University of Tromsø - The Arctic University of Norway, 9037 Tromsø, Norway; 2 Fram Centre, High North Research Centre for Climate and the Environment, 9007 Tromsø, Norway

**Keywords:** *Brucella pinnipedialis*, Epithelial cells, Hooded seal, Infection, In vitro, Marine ecosystem, Pinnipeds, Spillover

## Abstract

**Background:**

Marine *Brucella* spp. have been isolated from numerous pinniped and cetacean species, but pathological findings in association with infection with *Brucella pinnipedialis* in pinnipeds have been sparse. The capacity of brucellae to survive and replicate within host macrophages underlies their important ability to produce chronic infections, but previous work has shown that *B. pinnipedialis* spp. are rapidly eliminated from hooded seal (*Cystophora cristata*) alveolar macrophages.

**Results:**

To investigate if multiplication could take place in other hooded seal cell types, primary epithelial cells were isolated, verified to express the epithelial marker cytokeratin and challenged with three different strains of *B. pinnipedialis*; *B. pinnipedialis* sp. nov., *B. pinnipedialis* hooded seal strain B17, and *B. pinnipedialis* hooded seal strain 22F1. All strains were steadily eliminated and the amounts of intracellular bacteria were reduced to less than one-third by 48 h post infection. Intracellular presence was verified using immunocytochemistry.

**Conclusions:**

So far, intracellular multiplication in seal cells has not been documented for *B. pinnipedialis*. The lack of intracellular survival in macrophages, as well as in epithelial cells, together with the fact that pathological changes due to *B. pinnipedialis* infection is not yet identified in seals, suggests that the bacteria may only cause a mild, acute and transient infection. These findings also contribute to substantiate the hypothesis that seals may not be the primary host of *B. pinnipedialis* and that the transmission to seals are caused by other species in the marine environment.

## Findings

*Brucella* spp. were first isolated from marine mammals in 1994 [1] and were validly published as the species *Brucella pinnipedialis* sp. nov. and *Brucella ceti* sp. nov. in 2007 [2]. The bacteria have been isolated from numerous organs in pinniped and cetacean species, but pathological findings in association to infection with *B. pinnipedialis* in pinnipeds has only once been reported in eared seals (otariids) [3] and never, to date, in true seals. In dolphins, however, *B. ceti* have been shown to cause pathology in the central nervous system and the reproductive system [4, 5].

The capacity of brucellae to survive and replicate within host macrophages underlies their important ability to produce chronic infections [6], yet in vitro work has revealed that *B. pinnipedialis* hooded seal (HS) strain and *B. pinnipedialis* sp. nov. do not multiply in murine or human macrophage cell lines [7]. Brucellae are shown to exhibit a host preference [8], however, in vitro work with *B. pinnipedialis* HS strain*, B. pinnipedialis* sp. nov., *B. ceti* sp. nov., and *B. ceti* Atlantic white-sided dolphin (*Lagenorhynchus acutus*) strain in HS primary macrophages revealed no multiplication [9]. *Brucella* spp. may invade many cell types [10], but *B. pinnipedialis* HS strain and *B. pinnipedialis* sp. nov. were likewise rapidly eliminated from a human epithelial cell line [7]. The aim of the current study is to investigate whether *B. pinnipedialis* multiply in primary epithelial cells from HS.

The *Brucella* strains used were *B. pinnipedialis* sp. nov. (NCTC 12890^T^, BCCN 94-73^T^) [2] and two *B. pinnipedialis* HS isolates (spleen B17, and lung 22F1) [11]. The strains were kept and handled, and the final infective solutions were prepared, as previously described [7].

Epithelial tissue was collected from esophagus post mortem on two HSs (the same animals as previously described [9]). Approval of capture and import of animals was given by the appropriate authorities, and all animal use was in accordance with the Norwegian Animal Welfare Act and the regulations for use of animals in experimentation (permit no. 2402). Tissue cultures were prepared according to published protocols [12], and cultured in RPMI 1640, 10 % fetal bovine serum, 100 IU/ml penicillin, and 100 μg/ml streptomycin at 37 °C, 5 % CO_2_.

The epithelial origin of the cell culture was verified by immunocytochemistry using the epithelial marker cytokeratin. Adherent cells were fixed for 15 min at room temperature using 4 % paraformaldehyde (0.02 M sucrose, pH 7.2) and washed once in phosphate-buffered saline (PBS). Immune labeling was performed using mouse anti-pan cytokeratin antibody (PCK-26, Sigma–Aldrich, St. Louis, USA, 1:100). Secondary antibody was Alexa Fluor 546 goat anti-mouse IgG (Molecular Probes, Life Technologies, Paisley, UK, 1:500). For verification of intracellular bacterial localization, epithelial cells were challenged with *B. pinnipedialis* HS strain B17 as described in the gentamicin protection assay. Immune labeling was done using rabbit polyclonal anti-*Brucella* antibody, 1:100 (Prof. J.J. Letesson). Secondary antibody was Alexa Fluor 488 goat anti-rabbit IgG (Molecular Probes, 1:500). The fluorescent DNA dye DRAQ5 (Cell Signaling, Danvers, USA, 1:1000) was used for visualization of nuclei.

Primary HS epithelial cells were seeded (10^5^ cells/well) in 24 well plates and cultured for 8 days prior to infection. The cells were challenged as previously described for HeLa cells [7] and incubated for 1.5, 7, 24, and 48 h. Harvesting of cells and plating for evaluation of the number of intracellular bacteria were as previously described [7].

**Fig. 1:**
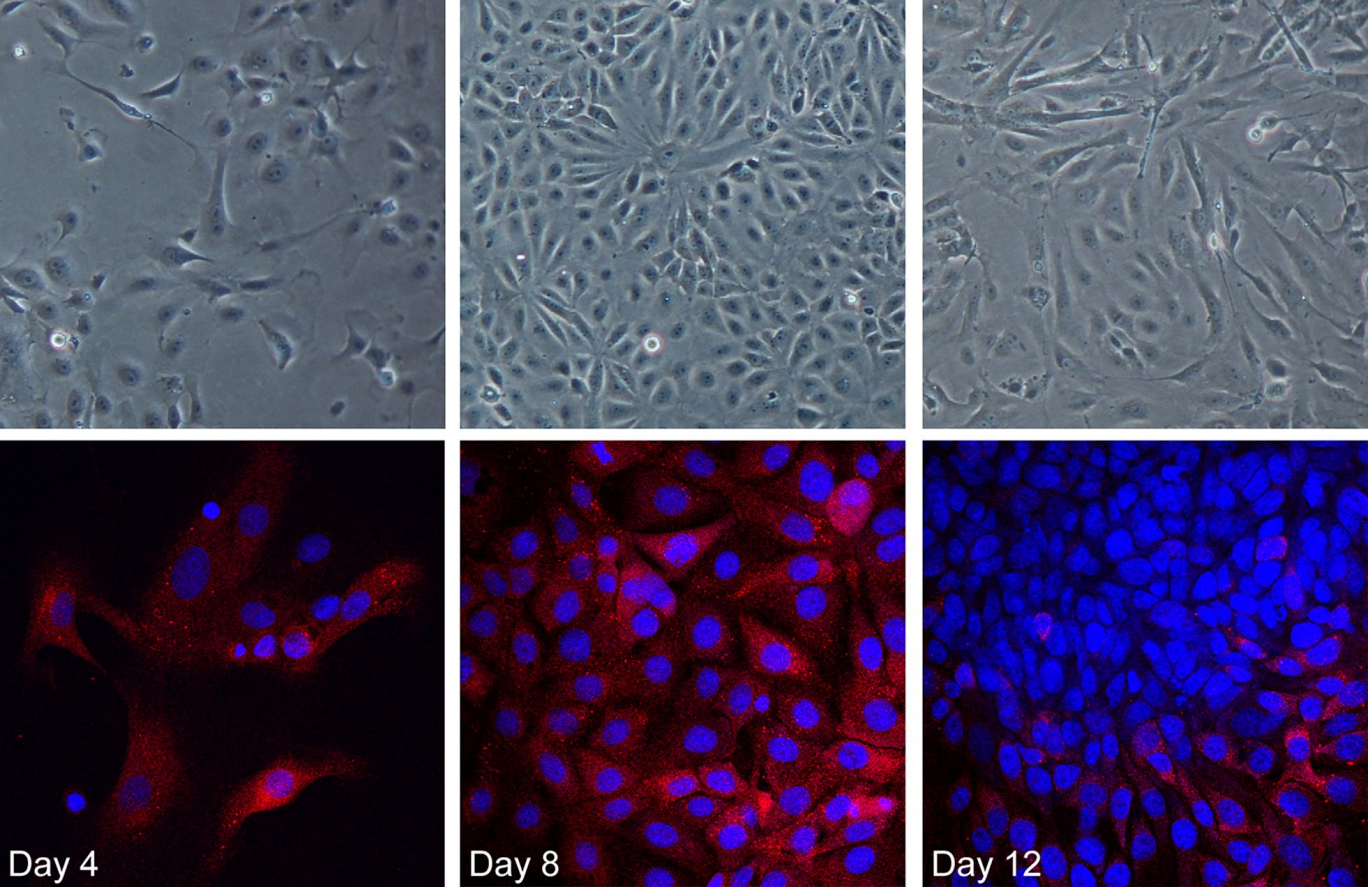
Verification of the identity of epithelial cells. Cultures of hooded seal primary epithelial cells staining positive for the epithelial marker cytokeratin (*red*). Images from confocal microscopy shown together with similar areas in light microscopy showing cell morphology at day 4, 8 and 12 after initiation of culture

After 8 days in culture the majority of the cells expressed the epithelial marker cytokeratin as illustrated by the anti-pan cytokeratin staining (Fig. 1). At day 12 the cultures contained a large amount of cells with a fibroblast-like morphology staining negative for anti-pan cytokeratin. Bacterial challenge was thus performed after 8 days in culture to ensure that the correct cell type was evaluated.

**Fig. 2:**
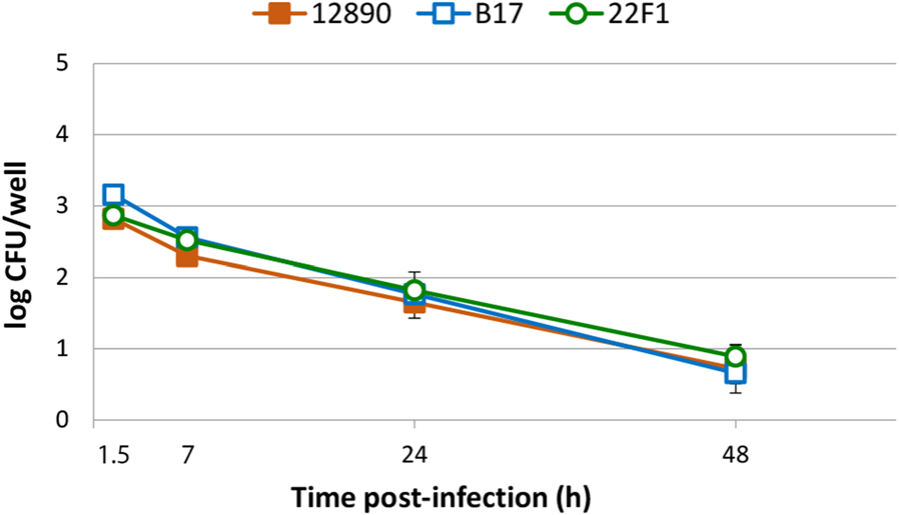
Survival of intracellular *Brucella pinnipedialis* in hooded seal epithelial cells. Hooded seal epithelial cells infected with *B. pinnipedialis* sp.nov. (12,890) and two different hooded seal isolates (B17 and 22F1) were lysed at 1, 7, 24 or 48 h after addition of gentamicin to determine the numbers of colony-forming units (CFU). Each *indicator* represents the mean of six parallels from two separate assays. *Error bars* correspond to the standard error of the mean

The results from the gentamicin protection assay revealed that all *B. pinnipedialis* strains were able to enter HS epithelial cells in vitro. When challenging the cells with a MOI of 500, *B. pinnipedialis* HS strains B17 and 22F1, and *B. pinnipedialis* sp. nov. showed moderate ability to enter, yielding log CFUs of 3.16, 2.87 and 2.82 at 1.5 h post infection (pi), respectively (Fig. 2). All three *B. pinnipedialis* strains were steadily eliminated and by 24 h pi the retrieved CFUs were reduced with 1.05–1.39 log CFUs. By 48 h pi, the amount of intracellular bacteria were reduced to less than one-third of the numbers of CFU at 1.5 h pi, yielding log CFUs of 0.66, 0.89 and 0.72. No significant differences (Student’s *t* test, *P* < 0.05 was considered significant) could be detected and the pattern of entry and elimination was similar for the three strains investigated (Fig. 2).

**Fig. 3:**
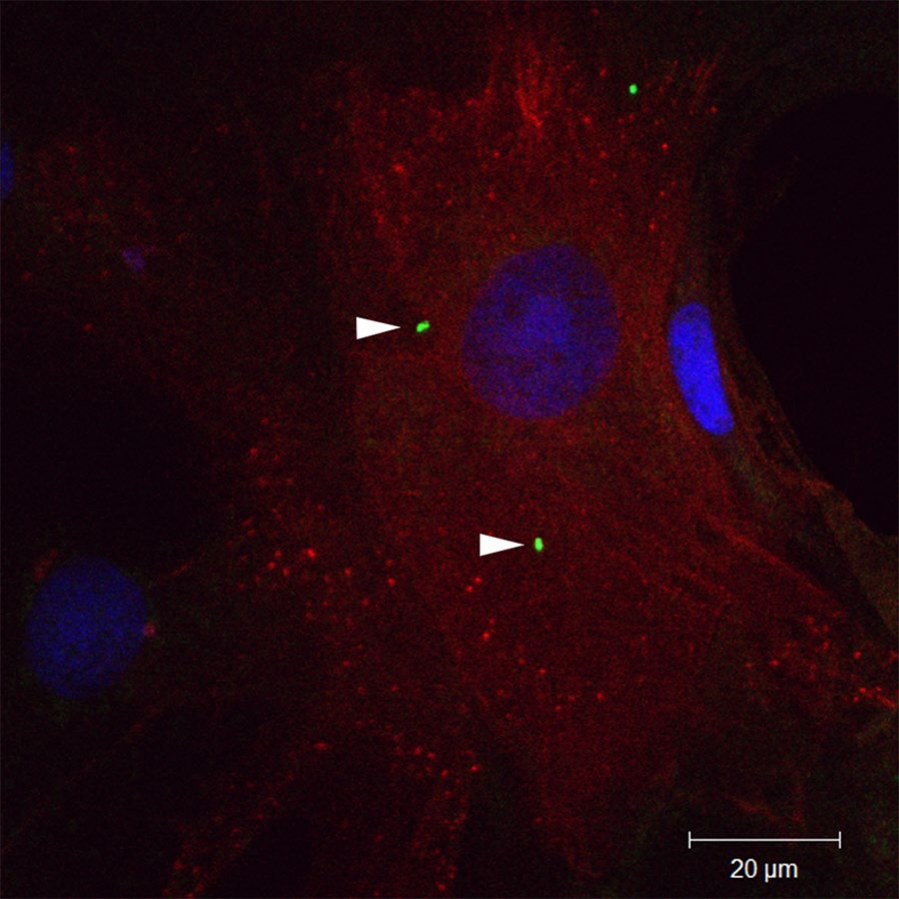
Intracellular localization of *Brucella pinnipedialis* hooded seal strain. Double immune labeling with anti-*Brucella* antibody (*green*) and anti-pan cytokeratin antibody (*red*) confirmed intracellular presence of hooded seal *B. pinnipedialis* strain B17 (*arrowheads*) in hooded seal epithelial cells. *Scale bar* 20 μm. The confocal image is a single optical section of 0.5 μm captured perpendicular to the Z-axis at the level of the nucleus using a Zeiss LSM510 META system (Carl Zeiss, Obercochen, Germany) equipped with a 40X 1.2NA water immersion lens

The intracellular localization of *B. pinnipedialis* HS strain B17 was confirmed by confocal microscopy (Fig. 3). Double immune labeling with anti-*Brucella* antibody and anti-pan cytokeratin antibody revealed intracellular presence of bacteria in cells staining positive for the epithelial marker.

For the first time, we present the results of infecting HS epithelial cells with *B. pinnipedialis* HS strain in vitro. Compiled with existing information from field research and in vitro macrophage infection assays, our results contribute to further understanding of marine *Brucella* infections in seals, a condition where epidemiology, pathogenesis and clinical importance are still unclear.

*Brucella pinnipedialis* HS strain seems to have a restricted, if any, ability to establish chronicity as the bacteria fail to multiply intracellularly in human and murine macrophages [7, 13]. The low pathogenicity of HS *B. pinnipedialis* has also been confirmed in a mouse model of infection [14]. Little information is available regarding the pathogenicity of these bacterial strains in seals, which are assumed to be the natural hosts of *B. pinnipedialis*, and the ability of the marine mammal brucellae to enter and multiply in host cells has been largely unexplored.

In addition to trophoblasts, which are target cells in the female reproductive organ, interaction with different cell types are shown for the pathogenic terrestrial brucellae. Macrophages are believed to be preferred as long time survival in the mononuclear phagocyte system of spleen, liver and bone marrow will sustain a chronic infection [6]. *Brucella pinnipedialis* HS isolate B17 and sp. nov. are previously shown to enter HS alveolar macrophages, but are rapidly eliminated [9]. As HSs are believed to be the primary host of *B. pinnipedialis* HS strain, it is intriguing that the HS isolate were not able to multiply in macrophages. In this work we aimed to explore if multiplication could take place in primary epithelial cells from a tentative host species, as shown for terrestrial pathogenic brucellae [15]. Epithelial cells would be the first cell type encountered given an exposure route through the food web, and both fish [16] or invertebrates, and possibly lungworms [17, 18], may be involved in transmission of marine brucellae. Although intracellular bacteria were not eliminated as quickly as reported for HS alveolar macrophages, the amount of viable intracellular bacteria steadily decreased during 48 h pi and no multiplication was detected. Entry of HS epithelial cells by *B. pinnipedialis* HS strain was verified by confocal microscopy, where intracellular bacteria were detected within cells staining positive for the epithelial marker (Fig. 3).

One can only speculate whether other cell types could be the target of *B. pinnipedialis* infection, supporting intracellular survival and multiplication. *Brucella abortus* is shown to survive within murine and human B-cells [19, 20] creating an intracellular niche that contributes to a chronic infection. Specific subpopulations of peripheral blood mononuclear cells (PBMCs) are not yet identified in the HS, however preliminary results indicate that *B. pinnipedialis* HS strain is quickly eliminated from infected HS PBMCs, reaching lysosomal compartments already at 1 h pi (Larsen, unpublished results). In light of the unusual high hematocrit found in hooded seals [21], erythrocytes could be a target for infection, as shown for *B. melitensis* in mice [22].

The lack of intracellular survival, together with the fact that pathological changes due to *B. pinnipedialis* infection is not yet identified in true seals, suggests that the bacteria cause a mild acute and transient infection. Given that *B. pinnipedialis* is unable to multiply intracellularly in macrophages and epithelial cells derived from other seal species, we argue that seals may not be the primary host for *B. pinnipedialis*, but rather a “dead-end” or spillover host being susceptible to infection transmitted from other hosts in the marine environment. Age-dependent serological and bacteriological patterns for *B. pinnipedialis* have been identified in HSs with a low probability of being positive for pups, a high probability for yearlings, followed by a decreasing probability with age, suggesting an environmental exposure post weaning during the first year of life followed by clearance of infection before the age of reproduction [23]. A similar age-dependent pattern of anti-*Brucella* antibodies was also identified in harbor seals [24, 25]. This raises the question of a reservoir of *B. pinnipedialis* in the food web, a hypothesis that is strengthened by the results presented herein and warrants further investigations.
